# Audiogenic Kindling Stimulation Fails to Induce Cerebral Overexpression of P-Glycoprotein and Limbic Crises in the GASH/Sal Model of Epilepsy

**DOI:** 10.3390/ijms27083377

**Published:** 2026-04-09

**Authors:** Laura Zeballos, Jerónimo Auzmendi, Alberto Lazarowski, Dolores E. López

**Affiliations:** 1 Instituto de Neurociencias de Castilla y León (INCYL), Universidad de Salamanca, 37007 Salamanca, Spain; laurazf@usal.es (L.Z.); jeronimo.auzmendi@gmail.com (J.A.); alazarowski@gmail.com (A.L.); 2 Instituto de Investigación Biomédica de Salamanca (IBSAL), 37007 Salamanca, Spain; 3 Departamento de Biología Celular y Patología, Facultad de Medicina, Universidad de Salamanca, 37007 Salamanca, Spain; 4 Instituto de Investigaciones Farmacológicas (ININFA, CONICET-UBA), Facultad de Farmacia y Bioquímica, Universidad de Buenos Aires, Junin 956, Buenos Aires C1113, Argentina; 5 Instituto de Fisiopatología y Bioquímica Clínica (INFIBIOC), Facultad de Farmacia y Bioquímica, Universidad de Buenos Aires, Buenos Aires C1417, Argentina

**Keywords:** audiogenic kindling stimulation, GASH/Sal hamsters, P-glycoprotein, seizure severity, spontaneous death

## Abstract

Experimental evidence indicates that a high seizure burden can induce cerebral overexpression of P-glycoprotein (P-gp) at the blood–brain barrier, a phenomenon associated with drug-resistant epilepsy under the “transporter hypothesis”, but also at the neuronal level, linked to a reduced seizure threshold, increased seizure severity (SS), status epilepticus (SE), and a high spontaneous death (SD) rate. In contrast, we recently described a progressive reduction in SS and the absence of SE and SD in GASH/Sal hamsters subjected to 45 audiogenic seizures. Here, we examined SS, SE, and the SD, and the expression of P-gp, erythropoietin receptor (*EPO-R*), hypoxia-inducible factor 1 alpha subunit (*HIF-1α*) and cyclooxygenase 2 *(COX-2*), in the brains of GASH/Sal hamsters following 20 audiogenic kindling stimulations (AUK-20). SS was evaluated using the midbrain and limbic severity scales; gene expression was assessed by RT-qPCR and P-gp protein levels were measured by immunohistochemistry and Western blot (IHC/WB) analysis. A modest decrease in midbrain SS was observed, without an increase in the already low limbic SS scores, and no SE or SD events occurred. P-gp levels remained low in both IHC and WB analyses. At the mRNA level, we detected increased *EPO-R* expression, decreased *HIF-1α*, and increased *COX-2* without an accompanying increased in *Abcb1b*. Unlike findings from other experimental epilepsy models, AUK-20 in GASH/Sal hamsters does not enhance limbic SS, trigger SE or SD, or induce P-gp overexpression in the brain. Independently of the implications for drug resistance, the lack of cerebral P-gp overexpression without increased SS in the AUK-20-GASH/Sal model supports a potential role of P-gp in modulating seizure severity and epilepsy-associated mortality risk.

## 1. Introduction

It is estimated that between 25 and 30% of patients with epilepsy fail to achieve adequate seizure control despite appropriate treatment with antiseizure medications (ASMs), ultimately developing refractory epilepsy (RE). First identified in cancer research, P-glycoprotein (P-gp) and other ABC-transporters (ABC-t) are directly associated with the multidrug resistant (MDR) phenotype [1]. The overexpression of these transporters at the blood–brain barrier (BBB) forms the basis of the so-called “transporter hypothesis”, one of the six most accepted hypotheses explaining refractoriness in epilepsy characterized by an MDR phenotype [2]. This hypothesis proposes that several factors, such as inflammation, hypoxia, the seizures themselves, and glutamic acid, contribute to the induction of P-gp overexpression in the brain, as demonstrated in several experimental models [3,4,5].

Although most available evidence associates P-gp with the mechanisms of drug resistance in RE, few studies have reported a possible relationship between neuronal P-gp expression, secondary to a high seizure burden, and greater seizure severity or an elevated risk of epilepsy-related death. In this regard, an initial study showed that repetitive seizures induced by 3-mercaptopropionic acid (3-MP) trigger marked cerebral overexpression of P-gp. This overexpression resulted in a progressive loss of protection by phenytoin (PHT), reaching 100% refractoriness after seven induced seizures (one per day), which was associated with generalized neuronal expression of P-gp [6]. Extending the duration of 3-MP-induced seizures showed that all animals developed an increased seizure severity (SS), and progressed to fatal status epilepticus on day 13, except for the group pre-treated with nimodipine, an inhibitor of P-gp activity [7]. Similar results were observed in a pentylenetetrazol (PTZ) kindling model, where, by day 7, fresh slides of the hippocampus exhibited strong P-gp expression associated with membranes depolarization, which normalized after nimodipine administration [8]. In both models, nimodipine (a P-gp inhibitor) restored seizure sensitivity and PHT concentrations in the hippocampus [8,9]. Furthermore, prolonged PTZ-induced seizure activity produced a progressive increase in SS as measured by the Racine scale, culminating in fatal status epilepticus on the tenth day of stimulation in all animals [10].

These findings, together with those reported by other authors confirmed in various experimental models and clinical studies, show that a high seizures burden induces brain P-gp overexpression associated with increased SS [11,12,13,14]. Moreover, experimental evidence indicates that even a single 20 min episode of status epilepticus (SE) is sufficient to induce P-gp expression in cortical pyramidal neurons and astrocytes. In vitro, excitotoxic stress (300 μM glutamate for 5 min) induced P-gp expression simultaneously with hypoxia-inducible factor 1α (HIF-1α) and the erythropoietin receptor (Epo-R) in primary cortical neurons. This suggested that both seizures and glutamate-mediated excitotoxicity activate a hypoxic response pathway [15].

Audiogenic kindling (AUK) is a well-established experimental model in epilepsy research that uses genetically epilepsy-prone rats (GEPRs) to induce seizures without drug administration, allowing the study of molecular and metabolic changes. In this model, exposure to acoustic stimuli induces generalized tonic–clonic seizures (GTCS), and repeated stimulation, through AUK, leads to the development of brainstem-triggered limbic seizures accompanied by cortical epileptiform activity [16]. Several studies have shown that repetitive sound stimulation in audiogenic models can result in spontaneous limbic-type seizures as a form of secondary epileptogenesis [16,17]. This secondary epileptogenesis typically affects the hippocampus and amygdala, and as these newly affected brain nuclei become involved, the severity of limbic seizures increases, while the severity of midbrain seizures decreases. AUK applied to GEPRs is therefore considered a reliable model for the development of temporal lobe epilepsy (TLE) [17]. Notably, approximately 70% of the patients with TLE are resistant to currently available ASMs, and overexpression of multidrug transporters has been reported in the surgically resected tissue from these patients [18,19,20]. Among the multiple changes associated with audiogenic kindling, several proteins play important roles, as they contribute to synaptic function [21], neurotransmitter transport [22], neuronal excitability [23,24], and signal transduction [25]. However, very little is known about alterations in brain P-gp expression and their relationship with SS, increased susceptibility to SE, or elevated spontaneous death rates in the AUK model. In this context, a single early study using GEPRs demonstrated that expression of the *Abcb1* gene, which encodes P-gp, was significantly increased in the cortex and midbrain 24 h after a single induced audiogenic seizure, with cortical expression remaining elevated 7 days after stimulation [26].

The “intrinsic severity hypothesis” of ASM drug resistance, summarized in Tang’s review [2], proposes that increased seizure severity and greater pharmacoresistance are consequences of the cumulative seizure burden, which is responsible for inducing this phenotype [27]. These observations align with the previously described increase in neuronal P-gp expression secondary to high seizure burden, which has been associated with more severe seizure phenotypes, higher resistance to ASMs, and a greater likelihood of spontaneous death following status epilepticus.

As a complement to the existing hypotheses of drug-resistant epilepsy, a role has been suggested for the neurotrophic receptor tyrosine kinase gene (*Ntrk2*) which encodes the tyrosine kinase receptor B (TrkB) involved in the development and maturation of the central nervous system. Enhanced activation of TrkB signaling has been suggested to promote epilepsy [28].

In sum, our previous experimental studies in rats [8,9,11] showed that high seizure load induces the overexpression of cerebral P-gp, resulting not only in greater drug resistance but also in an increase in SS, ultimately leading to episodes of status epilepticus accompanied by a high rate of spontaneous death. Consistent with these findings, an impaired GABA(A) receptor-mediated inhibitory pathway has been documented in both hippocampal kindling and in epilepsy following electrically induced SE in experimental models [29].

In contrast to these earlier results obtained in rat models of epilepsy, we recently reported a progressive loss of audiogenic seizure severity in GASH/Sal (Genetic Audiogenic Seizure Hamster, Salamanca) hamsters subjected to a sustained audiogenic kindling (GASH/Sal sAUK) protocol [30]. In this study, despite a high stimulus load, 45 audiogenic stimuli delivered over 15 days, the reduction in seizure severity became more pronounced as the number of stimuli increased [30]. Additionally, vesicular glutamate transporters 1 and 2 (VGLUT1 and VGLUT2), encoded by the *Slc17a7* and *Slc17a6* genes respectively, are commonly used as markers of glutamate release. In seizure-free GASH/Sal animals, under-expression of *Slc17a7* and *Slc17a6* mRNA has been reported in the cochlea compared with controls [31]. However, changes in the brain expression of these genes in GASH/Sal animals subjected to the AUK protocol have not yet been characterized.

Although antiseizure medications (ASMs) are highly effective in suppressing sound-induced seizures in GASH/Sal hamsters [32,33,34], no studies have examined the level of brain P-gp expression following exposure to a high burden of convulsive stimuli using the AUK protocol, nor its potential relationship with the SS index. 

This study aimed to investigate the relationship between the severity of seizures induced in the GASH/Sal epilepsy model subjected to audiogenic kindling and the expression levels of cerebral P-gp. Additionally, we sought to determine whether changes in the brain P-gp expression are associated with alterations in the expression of genes related to hypoxia, such as *HIF-1α* and *EPO-R*, as well as genes related to seizure-induced stress, such as *COX-2*. Furthermore, we aimed to assess whether the increased severity, particularly the onset of seizures, is linked to changes in the expression of genes implicated in neuronal hyperexcitability and neuroinflammation.

## 2. Results

### 2.1. Behavior

**Figure 1:**
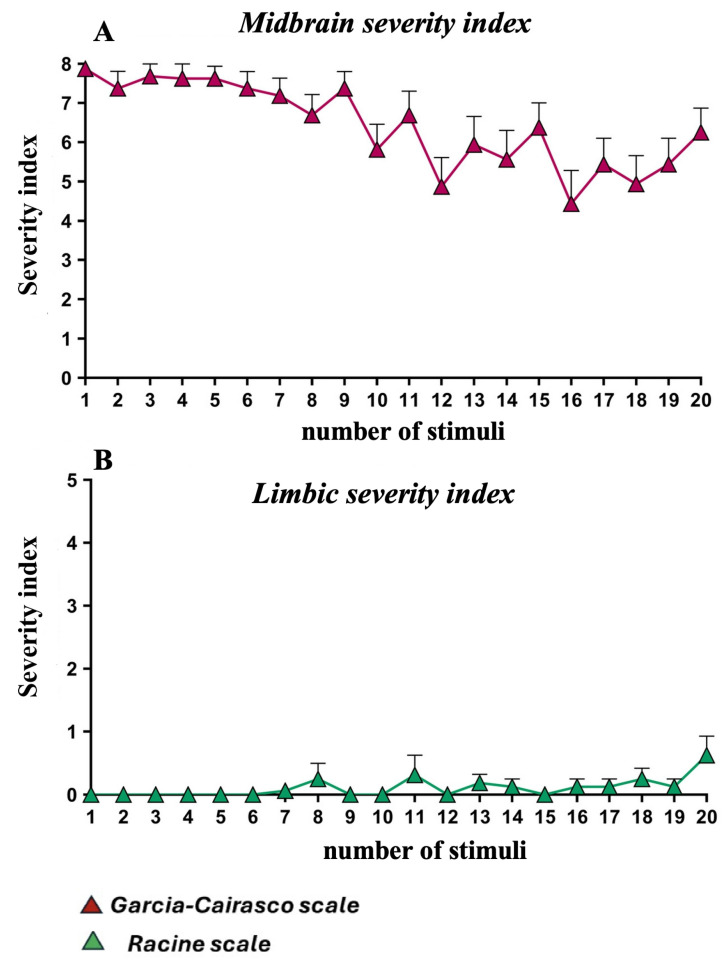
Seizure severity index (SSI) during administration of 20 audiogenic stimuli for 10 consecutive days. (**A**) Midbrain severity index scores assessed using the Garcia-Cairasco scale (▲). A slight reduction in SSI was observed from day 10 onwards, although midbrain seizure severity of the crises remained at an average value of 6. (**B**) Limbic severity index scores assessed using the Racine scale (▲). Across the 10-day AUK stimulation protocol, no increase in limbic SSI was detected in the same animals.

The AUK protocol, consisting of 20 repeated stimulations applied to GASH/Sal hamsters, produced a modest decrease in the midbrain seizure severity index (SSI), which ranged from a maximum of 7.88 ± 0.13 to a minimum of 4.88 ± 0.74 over a 10-day period (Figure 1A). Conversely, the limbic SSI did not increase at any point but instead remained consistently at minimal values, fluctuating between 0 and 0.63 ± 0.30 (Figure 1B).

Overall, these findings indicate that the 20-stimulation AUK protocol does not induce an increase in the severity of limbic seizures in GASH/Sal hamsters. 

### 2.2. Relative Expression of Synaptic and Neuroinflammatory Markers in Animals Subjected to the AUK Protocol

Because the SSI data obtained across the AUK protocol suggested no changes in limbic circuit activity in GASH/Sal hamsters, we next examined the relative expression levels of several biomarkers of synaptic function and neuroinflammatory activity in the hippocampus of AUK-stimulated animals.

**Figure 2:**
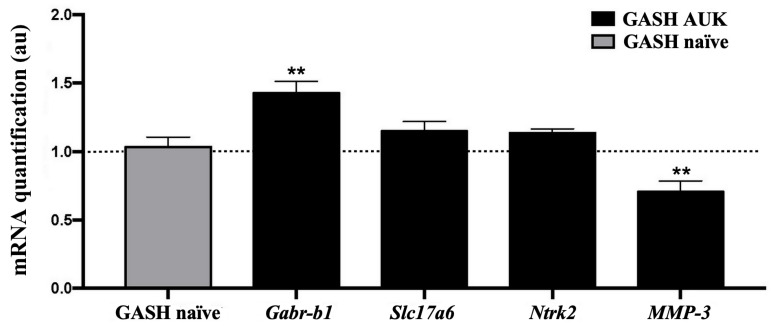
Differential expression of genes related to neuronal hyperexcitability and epileptogenesis in the hippocampus of GASH AUK hamsters compared with GASH naïve controls. The histogram shows the relative mRNA levels of *Gabr-b1*, *Slc17a6*, *Ntrk2*, and *MMP-3*. Gene expression values were normalized to *β-actin*. Each bar represents the mean ± SEM (n = 5 animals per experimental group). Asterisks indicate significant differences between groups (** *p* < 0.01). The dashed line represents the normalization level related to the different transcripts in GASH naïve hamsters. Abbreviations: *Gabr-b1*, beta 1 subunit of the GABA-A receptor; *Slc17a6*, vesicular glutamate transporter 2; *Ntrk2*, neurotrophin receptor tyrosine kinase 2; *MMP-3*, matrix metalloproteinase 3.

We observed a significant increase in the expression of the GABAergic receptor gene *Gabrb1* in the hippocampus of AUK hamsters (Figure 2). In contrast, mRNA levels of the matrix metalloproteinase 3 (*MMP-3*) gene were reduced in the hippocampus following stimulation. This metalloproteinase has been associated with inflammatory responses and aberrant synaptic reorganization through extracellular matrix degradation. Finally, no significant differences were detected in the gene expression of vesicular glutamate transporter 2, *Slc17a6*, or the neurotrophin receptor tyrosine kinase 2, *Ntrk2*. A graphical representation of these findings is provided in Figure 2.

### 2.3. mRNA Expression of the Abcb1 Isoforms and Inducer Genes in GASH/Sal Hamsters Following the AUK Protocol

**Figure 3:**
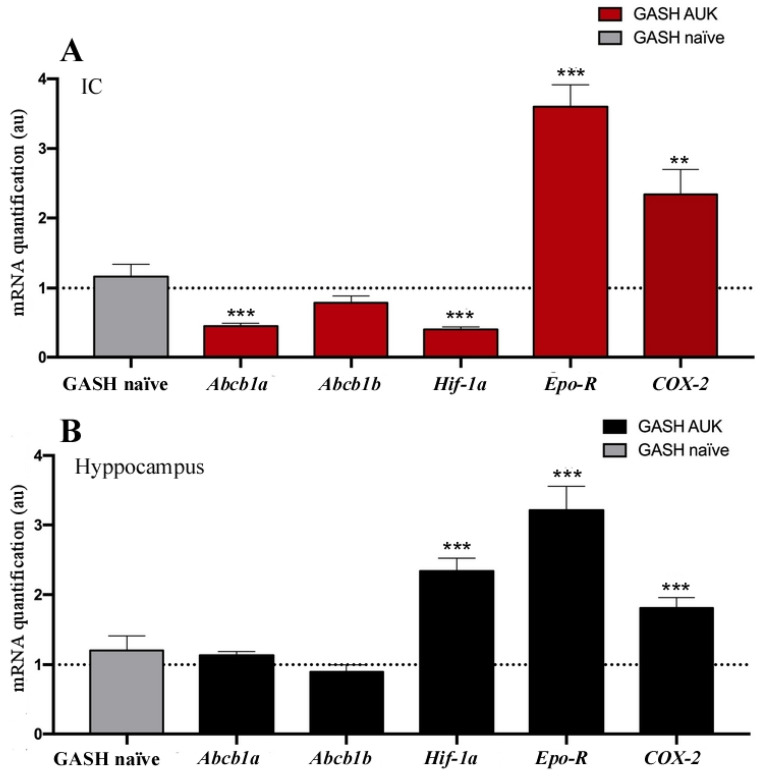
Differences in gene expression of P-glycoprotein-coding transcripts and transcripts related to its induction. Analysis was performed in (**A**) colliculus (■) and (**B**) hippocampus (■) of GASH AUK versus naïve GASH (■) hamsters. The histogram displays the relative mRNA levels of *Abcb1a*, *Abcb1b*, *Hif-1α*, *Epo-R*, and *COX-2.* Expression values for each gene were normalized to *β-actin*. Bars represent the mean ± SEM (n = 5 per experimental group for hippocampal analysis; n = 6 for GASH AUK and n = 5 for GASH naïve for IC gene expression analysis). Figure 3A: Differences detected in the IC between experimental groups. There is a decrease in *Abcb1a* and *HIF-1α* in the stimulated animals. In contrast, the levels of the *EPO-R* and *COX-2* transcripts were significantly higher in the IC following AUK stimulation. Figure 3B: Differences detected in the hippocampus between experimental groups. *HIF-1α*, *EPO-R*, and *COX-2* mRNA levels were significantly increased (*p* < 0.001) in the hippocampus of the AUK-stimulated group compared to the control animals. The dashed line represents the normalization level related to the different transcripts in GASH naïve hamsters. Asterisks indicate significant differences detected between experimental groups (** *p* < 0.01; *** *p* < 0.001). Abbreviations: *Abcb1a*: member 1a of the ABC transporter subfamily B; *Abcb1b*: ABC transporter subfamily B member 1b; *COX-2*: cyclooxygenase 2; *Epo-R*: erythropoietin receptor; IC: inferior colliculus; *HIf-1 α*: hypoxia-inducible factor 1 alpha subunit. In coordinates au: arbitrary units.

When analyzing gene expression in the inferior colliculus (IC) of markers associated with P-gp regulation, we observed a significant decrease in *Abcb1a* transcript levels in the AUK-stimulated animals (*p* < 0.001). In contrast, although *Abcb1b*, the constitutive (non-inducible) isoform, was present at lower levels in the colliculus in AUK animals, this difference did not reach statistical significance relative to non-convulsive controls. These changes were accompanied by a significant reduction in *HIF-1α* expression (*p* < 0.001). Conversely, the levels transcripts of *EPO-R* and *COX-2* were markedly increased in the IC following AUK stimulation (*p* < 0.001 and *p* < 0.01, respectively) (Figure 3A).

In the hippocampus, no significant differences were detected in the expression of either *Abcb1a* and *Abcb1b* genes, and notably, the inducible *Abcb1a* isoform did not increase following the 20-stimuli AUK protocol. As in the IC, hippocampal *EPO-R* and *COX-2* transcript levels were significantly elevated (*p* < 0.001) in the AUK-stimulated group compared with naïve animals. However, in contrast to the IC, *HIF-1α* gene expression in the hippocampus was also significantly increased after AUK stimulation (Figure 3B).

### 2.4. P-Glycoprotein Expression

We analyzed P-gp expression in both the IC and the hippocampus using immunohistochemistry and Western blots.

#### 2.4.1. Immunohistochemical Analysis of P-Glycoprotein

**Figure 4:**
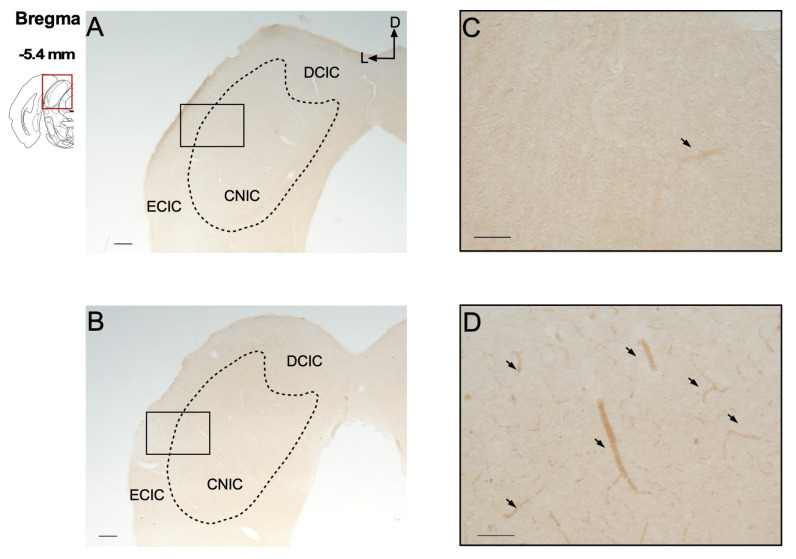
Comparison of the distribution of P-glycoprotein in the inferior colliculus. Schematic illustration of a coronal brain section indicating the corresponding Bregma level. (**A**,**B**) Immunohistochemical staining of P-glycoprotein in the inferior colliculus of the GASH naïve group (**A**) and the GASH AUK group (**B**). (**C**,**D**) Higher magnification images of the boxed regions in panels (**A**,**B**), respectively. In both naïve and AUK groups, P-gp immunoreactivity was restricted to blood vessels (arrows), with no detectable staining in other cellular elements of the IC. Scale = 200 μm (**A**,**B**); 50 μm (**C**,**D**). The dashed line delimits the different subdivisions of the inferior colliculus. Abbreviations: ECIC: external cortex; DCIC: dorsal cortex; CNIC: central nucleus of the inferior colliculus.

**Figure 5:**
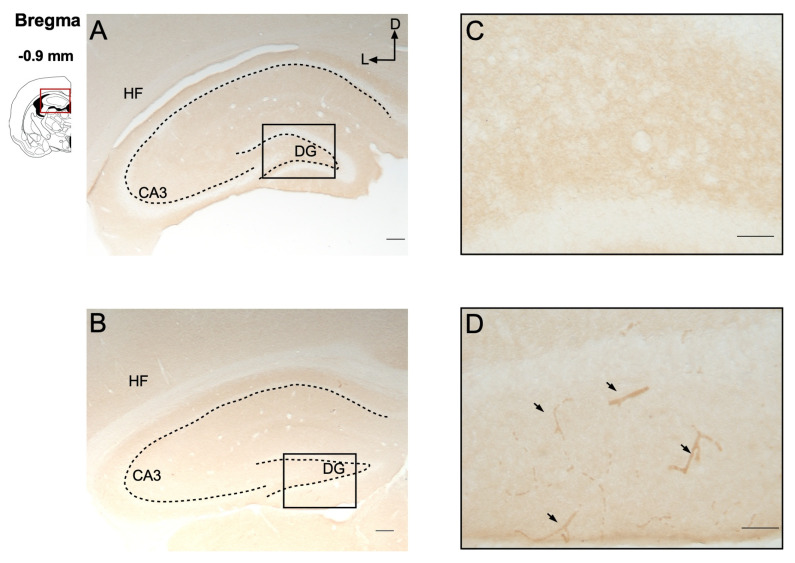
Comparison of the distribution of P-glycoprotein in the hippocampus. Schematic illustration of a coronal brain section indicating the corresponding Bregma level. (**A**,**B**) Immunohistochemical staining of P-glycoprotein in the hippocampus of the GASH naïve group (**A**) and the GASH AUK group (**B**). (**C**,**D**) Higher magnification images of the boxed regions in panels (**A**,**B**), respectively. Only a slight positive immunostaining was detected in the blood vessels (arrows) in the AUK GASH group. Scale = 200 μm (**A**,**B**); 50 μm (**C**,**D**). The dashed line delimits the different subdivisions of the hippocampus. Abbreviations: CA3: cornu ammonis area 3; DGDG: dentate gyrus; FH: hippocampal formation.

Immunohistochemical evaluation of both the IC and hippocampus revealed that P-gp expression was restricted to cerebral blood vessels within these regions (Figure 4 and Figure 5). No P-gp immunoreactivity was detected in other brain cell types, such as neurons, astrocytes, or microglia. Additionally, the repetitive seizures experienced by GASH/Sal hamsters following 20 AUK stimulations appeared to induce only a slight increase in vascular P-gp expression, without detectable expression in other cell types, in the epileptogenic regions of both the IC (Figure 4D) and the hippocampus (Figure 5D). These findings are consistent with the low limbic seizure severity values observed in this model.

#### 2.4.2. Western Blot of P-gp

**Figure 6:**
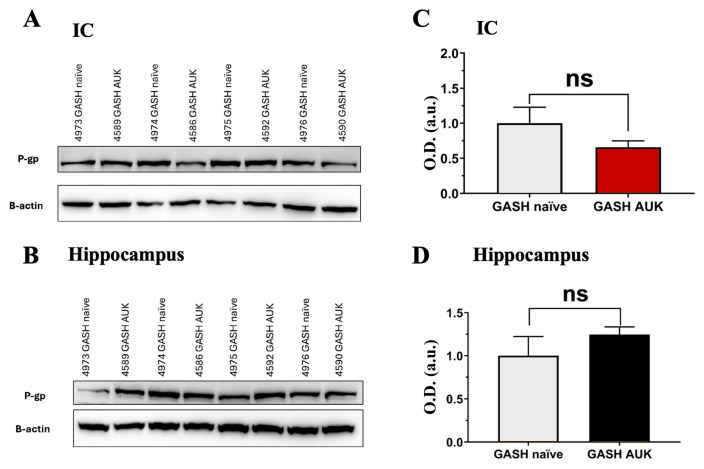
Western blot analysis of P-gp and ß-Actin in IC and hippocampus of GASH naïve and GASH AUK hamsters. Representative Western blots showing P-gp (170 kDa) and β-actin (42 kDa, loading control) in the IC (**A**) and in the hippocampus (**B**) of naïve and AUK-stimulated animals. (**C**,**D**): Densitometric quantification of P-gp immunoreactive bands from the IC (**C**) and hippocampus (**D**). Each bar represents the mean ± SEM (n = 4 per group), expressed as optical density (OD) values (in arbitrary units—a.u.) normalized to the corresponding β-actin band. Protein levels in the GASH naïve group were set to 1.0 for comparison. No differences in P-gp expression were detected in either brain region following the AUK protocol. WB of the GASH AUK group in the IC (■). WB of the GASH AUK group in the hippocampus (■). WB of the GASH naïve group (■).

In the GASH AUK group, Western blot detection of P-gp by immunoblotting in the IC showed a non-significant trend toward reduced protein levels compared with GASH naïve hamsters. In the hippocampus, no significant differences in P-gp expression were observed between groups (Figure 6). These findings are consistent with mRNA expression data (Figure 4).

## 3. Discussion

Repetitive AUK consisting of 20 stimulations produced only a slight decrease in midbrain SS (Garcia-Cairasco scale) [35] in GASH/Sal hamsters. However, this reduction was not accompanied by a progressive increase in limbic SS (Racine’scale) [36], which remained at minimum levels. This lack of increase in seizure severity (SS) in GASH/Sal hamsters clearly contrasts what has been observed in other species subjected to experimental status epilepticus induced by Li-pilocarpine or high convulsive loads with PTZ [37,38,39,40]. This divergent response indicates a lack of hyperexcitability or activity of pro-epileptogenic pathways in the hippocampus of GASH/Sal hamsters subjected to repetitive midbrain seizures, a finding entirely consistent with the low seizure severity index scores obtained in the Racine scale. Together, these results show that even a high load of audiogenic seizures in GASH/Sal hamsters does not induce neuronal P-gp expression, which is consistent with the lack of increase in limbic SSI.

### 3.1. Severity Indices and Limbic Recruitment

Previous studies have reported that as the severity of limbic seizures increases, the severity of midbrain seizures decreases [41,42]. However, this pattern was not observed in our research using GASH/Sal hamsters. In this model, the AUK protocol, consisting of a total of 20 stimuli, initially induces midbrain seizures with a high SSI, followed by a slight progressive decline over the 10-day experimental period. Notably, exposure to a substantially higher seizure load of three daily stimuli for 15 days (total of 45 stimuli) caused a pronounced decrease in seizure severity in GASH/Sal hamsters, ultimately reaching levels of minimal severity, as recently reported by our group [31]. Taken together, these data seem to indicate that at the mesencephalic level, the accumulation of seizure load induces a progressive protective mechanism that ultimately culminates in the near-complete absence of the stimulatory effect. The observed upregulation of *Gabr-b1* mRNA expression, along with decreased *MMP-3* mRNA levels, appear to support this trend toward enhanced protection.

In contrast, this protocol did not induce an increase in the severity of limbic seizures, which remained at minimal values throughout the experimental period. This finding suggests a lack of recruitment of hippocampal neurons into the firing mechanisms. Our data contradicts the results obtained in other genetic animal models of audiogenic seizures, in which repeated acoustic stimulation leads to the development of spontaneous limbic-type seizures, such as secondary epileptogenesis [43] affecting the hippocampus and amygdala [44,45,46], or with kindling protocols that show identical results [47].

Numerous repetitive-stimulation or kindling protocols have been described, varying according to the animal model employed, the method of induction, and the duration of exposure to proconvulsant stimuli. In this context, it was also demonstrated that daily audiogenic stimulations reproduce the key aspects of TLE development in Krushinsky–Molodkina (KM) rats, a strain genetically prone to audiogenic seizures (AGS). In KM rats, AUK stimulation induces abnormally high glutamate production, observed in aging KM rats, which may provide the basis for hyperexcitability of the hippocampus and increased seizure susceptibility in old age [48]. In this model, an increase in VGLUT2 was observed, accompanied by upregulation of extracellular signal-regulated kinases (ERKs) 1 and 2, cAMP response element-binding protein (CREB), and c-Fos in the hippocampus. Collectively, these results suggest that in KM rats, AUK stimulation activates glutamate production and synaptic vesicles with an increase in glutamate load [24].

Supporting the low limbic severity index results observed in our study, we did not detect significant differences in the transcripts levels of genes encoding VGLUT2 or TrkB proteins in AUK-stimulated hamsters. VGLUT2 has been associated with increased excitatory neurotransmission [49]. In parallel, increased expression of TrkB, a receptor for neurotrophins such as BDNF, has been associated with epileptogenic processes by activating MAPK signaling pathways [50]. More specifically, TrkB has been associated with mesial temporal lobe epilepsies, which in most cases are drug-resistant [28].

In contrast, the results obtained for the other hippocampal biomarker examined, metalloproteinase 3 (MMP-3), also contradict the overexpression described in the hippocampus in cases of temporal lobe epilepsy, which is associated with neuroinflammatory processes and neuronal hyperexcitability [51]. Furthermore, MMP-3 has recently been proposed as a robust predictive and diagnostic biomarker for epilepsy [52], with elevated levels reported in the hippocampus after kainic acid-induced seizures in mice [53], after pilocarpine-induced status epilepticus in rats [54], and also in childhood epilepsy [55]. In contrast to these observations, in our GASH/Sal AUK model, the expression levels of the *MMP-3* transcript were even lower than those detected in naïve hamsters. This reduction may reflect the activation of compensatory responses to the neuroinflammatory processes caused by repetitive seizures. Additionally, in this strain, plasma levels of MMP-3 were reduced following vagus nerve stimulation as compared with SHAM controls [56].

These findings, together with other results, suggest that GASH/Sal hamsters have biological responses distinct from those described in other rodent species. In this sense, it should be noted that GASH/Sal hamsters do not develop spontaneous seizures or status epilepticus despite repeated and accumulated seizures induction. This contrasts with what has been observed in several models of epilepsy in rats, whether induced by “lithium-pilocarpine” or “kainate” [57,58], or by audiogenic kindling, as in Wistar audiogenic rat (WAR) [59] and Krushinsky–Molodkina (KM) rats [17]. Furthermore, no cases of spontaneous death were observed in the GASH/Sal AUK-stimulated group, which does occur in many kindling models as above mentioned, and also described in DBA/2 mice [60,61]. 

Finally, we detected elevated levels of *Gabr-b1* transcripts in the hippocampus of the AUK-stimulated group, which could be associated with a protective mechanism against the high level of seizure burden experienced by the hamsters. Taken together, these findings allow us to speculate that an increase in inhibitory signaling is being attempted, potentially contributing to the absence of hyperexcitability in the hippocampus. The combined alterations in hippocampal gene expression, together with the absence of limbic seizures, indicate that this protocol is insufficient to recruit limbic structures in GASH/Sal hamsters. Nevertheless, it may promote compensatory inhibitory mechanisms in extra-mesencephalic structures. Alternatively, these results may point to the presence of an inhibitory mechanism in the hippocampus itself, offering protection against the possible development of secondary epileptogenesis.

### 3.2. Relationship Between Transporters and the Severity of Crises

Previous studies have shown that glutamate microinjection induces P-gp overexpression through the activation of *COX-2* [5]. In these experiments, rats injected with glutamate showed significantly higher levels of P-gp in the right hilum, located ventral to the injection site, compared with vehicle-injected controls. Notably, intracerebral microinjections of glutamate at nanomolar (subconvulsive) concentrations were sufficient to locally increase P-glycoprotein expression [62].

Another important aspect is that normal cerebral oxygenation can be reduced during or immediately after a seizure, particularly in the context of severe and repetitive seizures or status epilepticus. Ictal hypoxemia is a standard well-established phenomenon, most notably associated with tonic–clonic seizures, prolonged complex partial seizures, and reduced ASM administration [63,64,65]. These conditions generate a state of cerebral hypoxia–ischemia and activate the hypoxia-inducible factor 1 alpha (HIF-1α) [66,67], responsible for the induction of many genes, including *EPO-R* [68] and P-gp encoding gene [69,70]. Under any hypoxic condition, including seizures, HIF-1α is present in affected tissue as the main transcriptional regulator of cellular responses to oxygen deprivation. Thus, chronic hypoxia may be involved in the onset and development of epilepsy and increased susceptibility to seizures with the drug-resistant phenotype [71]. These observations support the hypothesis that P-gp induction by HIF-1α may occur as a direct consequence of the seizures themselves [72]. 

Our present results in the GASH/Sal model seem to indicate a P-gp and *HIF-1α* expression pattern opposite to that previously described by our group [15,73] and by other authors [74,75] in different experimental epilepsy and kindling models in rats. Despite the high seizure burden and the increase in *COX-2* (an inducer of P-gp), there was no increase in *Abcb1b* (mRNA) and Pg-p (IHC/WB). This suggests that the increase in *COX-2* alone was insufficient to induce P-gp expression in the absence of hypoxic conditions in this model. This interpretation is further supported by the reduction in *HIF-1α* mRNA. These results imply that GASH/Sal hamsters do not develop a post-convulsive hypoxic response, and that, under these conditions, *COX-2*-dependent induction of P-gp induction is not activated.

We could also speculate that in this AUK model, the absence of cerebral P-gp overexpression could prevent the development of P-gp-dependent drug-resistant epilepsy (as “transporter hypothesis” indicates), although this was not demonstrated in this study. However, the lack of P-gp positive neurons in the hippocampus may help to explain the unchanged severity of limbic seizures despite the high cumulative convulsive load. Furthermore, the increase in *EPO-R* transcripts in the absence of increased *HIF-1α* expression could be a consequence of its induction mediated by transcription factors other than HIF-1α. Indeed, cerebral expression of EPO-R is known to be regulated by *HIF-2*, *GATA-1/GATA-2*, *Sp1*, *NF-kB* and *STAT5* [76,77].

P-gp plays a central role in the “transporter hypothesis” of drug-resistant epilepsy [2,78,79,80]. More recently, its direct involvement in facilitating seizures has also been proposed. In brief, aberrant neuronal expression of P-glycoprotein may lower the seizure threshold, thereby promoting the development of new seizures, and cardiac dysfunction associated with increased rates of sudden death [81,82]. This severe bradycardia scenario could trigger a feedback loop in which repetitive seizures lead to an increase in P-gp expression in the BBB, reinforcing pharmacoresistance, while simultaneously inducing its expression in neurons. Neuronal P-gp expression may then facilitate membrane depolarization and the development of more severe and frequent seizures, which is in line with the intrinsic severity hypothesis [2,27].

In contrast to these reports, our current study shows that repetitive stimulation in GASH/Sal hamsters does not lead to more severe seizures, nor does it affect other brain structures as previously documented [46,83,84]. The data above seem to indicate that the role of P-gp in the GASH/Sal epilepsy model differs substantially from that described in other audiogenic epilepsy models. For example, while *Abcb1a* is overexpressed in the Wistar audiogenic rat (WAR), no statistically significant differences in *Abcb1a* expression have been observed between seizure-prone GASH/Sal hamsters and their control counterparts [85]. Furthermore, the absence of mortality in this model could indicate that their seizures were, in some way, contained, without systemic consequences. These data are consistent with the results obtained from the analysis of the significantly low gene expression of *Abcb-1* family transcripts in the colliculus and hippocampus, and the absence of P-gp immunostaining in these same structures.

In conclusion, our results indicate that AUK stimulation induces a clear differential response in GASH/Sal hamsters, characterized by an extremely low limbic seizure severity index, no status development, no spontaneous death, and a lack of induction of neuronal P-gp expression.

This response contrasts sharply with the outcomes reported in other rodent models subjected to a high seizure load. Taken together, these findings support the notion that neuronal P-gp expression plays a critical role in the intrinsic severity hypothesis of refractory epilepsies.

## 4. Materials and Methods

### 4.1. Animals

For this study, we used the Syrian hamster strain *Mesocricetus auratus*, GASH/Sal (Genetic Audiogenic Seizure Hamster, Salamanca, Spain), provided by the Animal Experimentation Service of the University of Salamanca (S.E.A.). Male animals between two and four months of age were used. All animals were housed in the animal facility of the Institute of Neurosciences of Castilla y León, with ad libitum access to food and water, and maintained at a temperature of 21 ± 2 °C, with 12 h light and dark cycles. All experimental procedures were conducted in accordance with Spanish regulations governing the use and care of laboratory animals established in Royal Decree 53/2013 and Order ECC/566/2015 of March 20 and were approved by the Bioethics Committee of the University of Salamanca. A total of 20 GASH/Sal hamsters were assigned to the audiogenic kindling (AUK) group, receiving two stimulations per day (GASH AUK group) for 10 consecutive days, for a total of 20 audiogenic stimulations. An additional control group of 10 GASH/Sal hamsters with no stimulation (GASH naïve) was included. These classified group assignments according to the stimulation protocol are summarized in Table 1.

### 4.2. AUK Protocol

An audiogenic kindling (AUK) protocol was used, in which experimental animals received two acoustic stimulations per day for 10 consecutive days, for a total of 20 stimulations. Each induced seizure was recorded and subsequently analyzed using both the Racine scale [36] and the Garcia-Cairasco [35] scale.

GASH/Sal hamsters were assigned to two groups: an untreated control group (GASH naïve) and a group subjected to the AUK protocols (AUK group). In both groups, P-gp expression was examined by immunohistochemistry in fixed brain sections and by RT-qPCR analysis of transcripts in samples of fresh brain homogenates. We also evaluated genes known to participate in the induction of P-gp expression, including *HIF-1α* and *COX-2*, which encode hypoxia-inducible factor 1 *α* subunit and cyclooxygenase 2, respectively. In addition, we assessed the relative expression of the erythropoietin receptor given its relevance as a marker of hypoxia. In animals exposed to the kindling protocol, behavioral seizure severity was assessed throughout the 10-day stimulation period.

**Figure 7:**
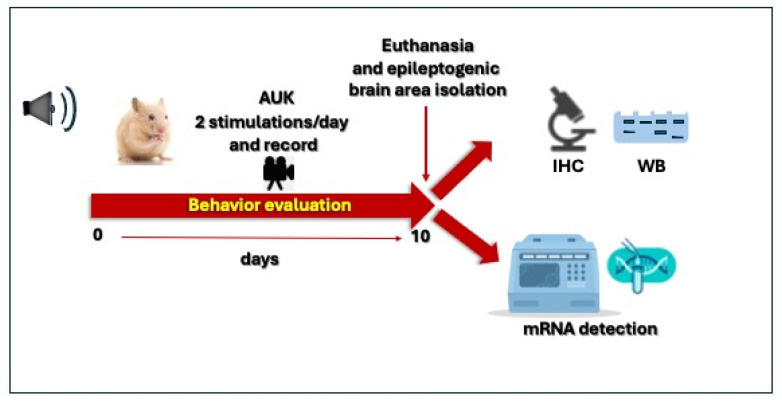
Schematic representation of the AUK protocol applied to GASH/Sal hamsters.

Finally, to validate the role of the hippocampus in seizure responses to AUK, we analyzed several molecular markers associated with neuronal hyperexcitability or neuroinflammation, which could indicate significant changes in the hippocampus. These included the vesicular glutamate transporter, VGLUT2, encoded by the *Slc17a6* gene; the neurotrophin receptor, TrkB, encoded by the *Ntrk2* gene; metalloproteinase-3, *MMP-3*; and the beta-1 subunit of the GABA-A receptor, encoded by the *Gabr-b1* gene. The experimental design is shown in Figure 7.

### 4.3. Audiogenic Stimulation

The animals were placed individually in a methacrylate sandpit and allowed to acclimate for one minute. They were then exposed to the auditory stimulus, consisting of continuous white noise spanning 0 to 18 kHz at an intensity of 115 to 120 dB. Exposure continued until the start of the running phase, or if running did not occur, for a maximum duration of one minute. Following stimulation, the animals remained in the sandpit for an additional one-minute post-stimulus period. AUK stimulations were administered twice daily, at approximately 9:00 AM and 7:00 PM. The recordings of each session were used to evaluate both brainstem and limbic seizure severity indices (SSI).

### 4.4. Seizure Severity Evaluation

The severity index (SSI) for each induced seizure was assessed using both mesencephalic and limbic severity scales. Limbic seizure severity was evaluated using the Racine scale [36], which ranges from 0 (no seizure activity) to 5 (maximum severity; Table 2). Midbrain seizure characteristics were analyzed using the Garcia-Cairasco scale [35], which scores seizure behaviors from 0 to 8 according to the behaviors observed during the stimulus (Table 3). The behavioral criteria associated with each score for both scales are summarized in Table 2 and Table 3.

### 4.5. Euthanasia and Sample Collection

For the molecular analyses, the animals were euthanized, and fresh samples were collected. The hamsters were anesthetized with CO_2_ and, once areflexia was confirmed, they were decapitated. The brain was rapidly removed from the skull, and the regions of interest were dissected immediately. All collected samples were placed into labeled Eppendorf tubes, rapidly frozen by immersion in liquid nitrogen, and stored at −80 °C until further processing.

### 4.6. Perfusion

For immunohistochemical analyses, four animals were perfused 90 min after the final AUK stimulus. Hamsters were anesthetized with a lethal intraperitoneal injection of sodium pentobarbital (60 mg/kg). Once areflexia was confirmed, the thoracic cavity was opened to expose the diaphragm and then the heart. Transcardiac perfusion was subsequently performed following the routine protocol established in our laboratory [86]. After perfusion was confirmed, the animals were decapitated, the skull was opened, and the brain was extracted. Brain samples were post-fixed in 4% paraformaldehyde (PFA) in phosphate buffer (PB) at 4 °C for 24 h. After fixation, laterality was determined by inserting an entomological needle into the left hemisphere. The tissue was then cryoprotected by immersion in 30% sucrose in PB for 48 h at 4 °C.

### 4.7. Gene Expression Analysis

Hippocampal and inferior colliculus samples were collected from GASH/Sal naïve animals and from those subjected to the AUK protocol. These tissues were used for gene expression analyses by RT-PCR methods.

### 4.8. mRNA Extraction and Purification

For comparative gene expression analysis, we extracted RNA from each tissue sample using the TRIzol^TM^ extraction method according to the manufacturer’s protocol. Briefly, each dissected structure was homogenized in the appropriate volume of TRIzol^®^ (ThermoFisher Scientific, Waltham, MA USA) adjusted to tissue weight. The homogenate was centrifuged at 12,000× *g* for 5 min at 4 °C. The resulting supernatant was incubated for 5 min at room temperature, after which chloroform was added. This mixture was incubated for an additional 3 min at room temperature and centrifuged at 12,000× *g* for 15 min to allow phase separation.

The aqueous phase containing RNA was transferred to a clean tube, mixed with isopropanol, and incubated for 10 min. Samples were then centrifuged at 12,000× *g* for 10 min at 4 °C. The supernatant was discarded, and the RNA pellet was washed with 75% ethanol. Following centrifugation at 7500× *g* for 5 min, the resulting pellet was air-dried and resuspended in Milli-Q water. The RNA concentration and purity were determined by spectrophotometry at 260 nm (Nanodrop™ 2000C spectrophotometer, Thermo Scientific, Waltham, MA USA). For reverse transcription of the RNA into complementary DNA (cDNA), we used the RevertAid First Strand cDNA Synthesis Kit (ThermoFisher Scientific, Waltham, MA USA). For each sample, RNA was mixed with Oligo (dT)18 primers and incubated for 5 min at 65 °C. To this mixture, a buffer solution consisting of reaction buffer, Ribolock RNase Inhibitor, dNTPs, and the reverse transcription enzyme, ReverAid M-MulV, was added. We then incubated the new sample for 60 min at 42 °C and 5 min at 70 °C. The resulting cDNA was stored at −20 °C until further use.

### 4.9. RT-qPCR Technique

Gene expression levels were quantified using real-time quantitative polymerase chain reaction (RT-qPCR). Specific primer pairs were designed for the amplification of the different genes of interest and the housekeeping gene (β-actin).

In humans and dogs, the *ABCB1* gene is present as a single locus, whereas in contrast, most rodents possess two *Abcb1* genes encoding the isoforms: *Abcb1a* and *Abcb1b* [87]. The presence of distinct accession numbers for “Hamster Abcb1a” and “Hamster Abcb1b” (NM_001243988 and NM_001243989, respectively) confirms this duplication in the Syrian hamster genome [88]. Orthology is highly conserved between mouse and Syrian hamsters’ *Abcb1* gene: sequence identity > 85–90% is consistent with the *Abcb1a* locus, while a second sequence with a slightly lower identity (~80–85%) most likely corresponds to *Abcb1b*. Sequences of all genes of interest were obtained from *Mesocricetus auratus* entries in the Ensembl database (https://www.ensembl.org/index.html, accessed on 4 November 2025). Primers (18–24 base pairs) spanning exon–exon junctions were designed using the Primer3 v.0.4.1 software program (https://bioinfo.ut.ee/primer3-0.4.0/, accessed on 10 September 2025). Annealing temperatures and potential secondary structures were evaluated using the NetPrimer (PremierBioSoft; http://www.premierbiosoft.com/NetPrimer/AnalyzePrimer.jsp, v. 1.10, accessed on 10 September 2025) to ensure primer stability. Finally, the NCBI Primer Blast database (https://www.ncbi.nlm.nih.gov/tools/primer-blast/ accessed on 5 October 2025) was used to verify specific amplification. The primer sequences used in this study are listed in Table 4.

RT-qPCR was performed by using a SYBR Green-based fluorescence detection method (Power SYBR™ Green, Thermo Fisher Scientific, Cat. No. 4367659). Each cDNA sample was analyzed in triplicate. Reaction mixtures (20 µL total volume) contained the required amount of cDNA template, 7 µL of SYBR Green Master Mix, 0.4 µL of each primer (10 µM), and nuclease-free water to volume. Amplification was carried out using a QuantStudio^TM^ 7 Flex Real-Time PCR System (Applied Biosystems, Waltham, MA, USA) under the following conditions: i initial denaturation at 95 °C for 10 min, followed by 40 cycles of 15 s at 95 °C and 30 s at 60 °C. For each gene, samples from at least four animals per group were analyzed in triplicate. For data normalization, β-actin was used as the reference gene due to its constitutive and stable expression across tissues and treatments [89]. The results were analyzed using the 2^−ΔΔC_T_^ method [90], which allows for the relative quantification of changes in gene expression. The cycle threshold (C_T_) was defined as the point at which fluorescence increased above background levels, reflecting the exponential phase of target amplification. Fold changes values represent expression differences between experimental and control conditions after normalization to the reference gene [90]. The calculations were performed as follows: 2^−ΔΔCT^, where ΔC_T_ = C_T,target_ − C_T,reference gene_, and ΔΔC_T_ = ΔC_T,target_ − ΔC_T,calibrator_. Primer efficiencies were determined using the equation E = 10^[−1/slope]^.

### 4.10. Western Blot

Protein extraction from the inferior colliculus and hippocampus of naïve and AUK-stimulated GASH/Sal animals was performed using radioimmunoprecipitation assay buffer (RIPA, Cell Signaling Technology, Danvers, MA, USA, Cat. No. 9806S) supplemented with a protease inhibitor cocktail (Thermo Fisher Scientific Inc., Waltham, MA USA, Cat. No. 78442). Tissue homogenates were centrifuged at 14,000× *g* for 15 min, and protein concentrations of the resulting supernatant were determined using the Bio-Rad DC Protein Assay Kit (Bio-Rad, Hercules, CA, USA, Cat. No. 500-0116).

Protein samples were denatured by heating at 95 °C for 5 min in the presence of 10× NuPAGE™ Sample Reducing Agent (Thermo Fisher Scientific Inc., Cat. No. NP0004,) and 4× NuPAGE™ LDS Sample Buffer (Thermo Fisher Scientific Inc., Cat. No. NP0007). Equal amounts of protein (30 µg for the inferior colliculus and 60 µg for the hippocampus) were loaded onto Bolt™ 10% Bis-Tris polyacrylamide gels. Electrophoresis was carried out at room temperature, starting at 90 V for 30 min, followed by 150 V until completion.

Proteins were transferred to nitrocellulose membranes using the iBlot™ 2 dry transfer system. To block non-specific binding, membranes were incubated for 1 h at room temperature in 5% non-fat dry milk prepared in Tris-buffered saline with 0.1% Tween-20 (TBS-T). Membranes were then incubated overnight at 4 °C with a rabbit polyclonal anti-P-glycoprotein primary antibody (Abclonal, Woburn, MA, USA, Cat. No A11747) diluted 1:1000 in TBS-T. After primary incubation, membranes were washed with TBS-T and incubated for 2 h at room temperature with a horseradish peroxidase (HRP)-conjugated goat anti-rabbit secondary antibody (Bio-Rad, Cat. No. 1706515) diluted 1:3000. Protein bands were visualized using a chemiluminescent substrate, and signals were detected using a chemiluminescent system comprising the MicroChemi imaging unit and GelCapture software v 7.0.18. To verify equal protein loading, membranes were stripped, re-blocked, and incubated with a monoclonal anti-β-actin antibody (Santa Cruz Biotechnology, Dallas, TX, USA, Cat. No. sc-47778) at 1:1000 dilution for 1 h at room temperature, followed by incubation with an HRP-conjugated goat anti-mouse secondary antibody (Bio-Rad, Cat. No. 1706516). Chemiluminescent detection was performed as described above. Band intensity was quantified by densitometric analysis using ImageJ software (v.1.53C), and the signal for each target protein was normalized to its corresponding β-actin signal. Data are expressed in arbitrary densitometry units.

### 4.11. Immunohistochemistry: Floating Sections Procedure

Perfusion-fixed, cryoprotected brain samples were placed in a coronal mold and trimmed to define the region of interest. Tissue was sectioned coronally at a thickness of 40 µm using a freezing microtome. Serial sections were placed into six 6-well plates containing phosphate buffer (PB) and processed according to standard immunohistochemical procedures established in our laboratory [91]. Briefly, sections were washed repeatedly in PB and incubated in a solution to inhibit endogenous peroxidase activity. Non-specific binding was blocked by incubation in 2% heat-inactivated fetal bovine serum for 1 h at room temperature. Sections were then incubated for 72 h at 4 °C with a rabbit monoclonal anti-P-glycoprotein primary antibody (1:250, Abcam, Cambridgeshire, UK, Ct. No ab170904). After, sections were washed and incubated for 2 h at room temperature with a biotinylated goat anti-rabbit secondary antibody (1/200, Vector Laboratories, Newark, CA, USA, Cat. No. BA-1000). Following secondary incubation, the avidin–biotin complex (ABC) complex was applied for 90 min (VECTASTAIN^®^ ABC-HRP Kit, Vector Laboratories, Cat. No. PK-4000). Immunolabeling was visualized using 3,3-diaminobenzidine tetrahydrochloride (DAB) chromogen (ImmPACT DAB Peroxidase Substrate, Vector Laboratories, Cat. No. SK-4105) under visual and microscopic control. After the reaction was complete, sections were washed, mounted onto slides, dehydrated, cleared, and coverslipped for microscopic examination.

### 4.12. Statistical Analysis

Statistical analyses were performed using GraphPad Prism, version 8 (GraphPad Software, San Diego, CA, USA). Gene expression differences between groups were assessed using the Student’s *t*-test, and results were considered statistically significant at *p* < 0.05 (*p* < 0.05 [*], *p* < 0.01 [**], and *p* < 0.001 [***]).

## 5. Conclusions

The AUK protocol induces severe midbrain seizures in GASH/Sal hamsters but does not elicit limbic seizures. No evidence of hippocampal hyperexcitability or activation of pro-epileptogenic pathways was detected in animals subjected to repeated midbrain seizures. Taken together, these findings suggest the activation of adaptive mechanisms that mitigate the effects of midbrain seizures in this model.

The AUK protocol does not induce an increase in brain P-gp expression, despite the marked upregulation of *COX-2*, which coincides with a decrease in *HIF-1α* expression. This pattern indicates the absence of a post-convulsive hypoxic response, an outcome that contrasts sharply with other rodent epilepsy, where seizures induce P-gp overexpression and which is associated with an increase in seizure severity.

The lack of P-gp overexpression without increased seizure severity strongly supports a potential functional role for neuronal P-gp within the framework of the intrinsic severity hypothesis of refractory epilepsy.

## Figures and Tables

**Table 1:** Experimental animals used for the control and AUK groups. The table summarizes the stimulation protocol applied to each group, the corresponding group designations, and the number of animals included per experimental condition.

Group	Stimulation Protocol	Nº
GASH naïve	No stimulation	10
GASH AUK	Audiogenic kindling, 2 stimulations/day for 10 days	20

**Table 2:** Limbic severity indices (Racine, 1972) [36].

Behavior	SSI
No seizure	0
Orofacial movements	1
Head myoclonus and/or severe orofacial movements	2
Foreleg myoclonus	3
Foreleg clonus with elevation	4
Generalized tonic–clonic seizures with loss of postural control	5

**Table 3:** Midbrain severity indices. Adapted from Garcia-Cairasco et al., 1996 [35].

Behavior	SSI
No seizure	0
One run and turn	1
One run and turn, followed by atonic hopping and falling	2
Two (or more) runs and turns followed by atonic hopping and falling	3
All the above behaviors plus tonic seizures with back arching	4
All the above behaviors followed by partial and/or generalized clonic seizures	5
All the above behaviors followed by ventroflexion of the head	6
All the above behaviors followed by hyperextension of the forelimbs	7
All the above behaviors followed by hyperextension of the hindlimbs	8

**Table 4:** Primers used for gene amplification in RT-qPCR and amplicon size.

Gene	Sequence (5′-3′)	Amplicon (bp).
*Gabr-b1*	CGAGAGAGTTTGGGGCTTCT	219
	CTTCACTTCCGAGACCATGTC	
*Slc17a6*	AGTCATTGCGATGCCCTTAG	204
	CCTAACAGGTTTGCGCTCTC	
*Ntrk2*	GGAGACTACACACTAATGGC	199
	CAGCAACATCTGTAGAAGGGA	
*MMP-3*	CCGTGATACCCACCAAATCT	185
	GGGCCAAAATGAAGAGATCA	
*Abcb1a*	GGAAATCATTGGGGTGGTGA	127
	GGCATTGGCTTCCTTGACAG	
*Abcb1b*	CTGGTATGGGACCTCCTTGG	122
	AGGCTTCTATGTTTGGGGCG	
*HIf-1 α*	ATCAGTTGCCTCTTCCCCAC	181
	ACCATAACGAAGCCATCCAGG	
*EPO-R*	CTATGACCACCCACATCCGC	115
	CGCAGGTTGCTCAGAACACA	
*COX-2*	GCTTACAAGACGCCACATCA	224
	TCGTAAAGACGGTAGGGCAA	
*B-act*	AGCCATGTACGTAGCCATCC	115
	ACCCTCATAGATGGGCACAG	

Abbreviations of amplified genes: *Abcb1a*: ATP binding cassette, subfamily B, member 1, isoform a; *Abcb1b*: ATP binding cassette, subfamily B, member 1, isoform b; *β-act*: beta actin; *COX-2:* cyclooxygenase 2; *EPO-R:* erythropoietin receptor; *Gabr-b1*: beta 1 subunit of the GABA-A receptor; *H1f-1 α*: hypoxia-inducible factor 1 alpha subunit; MMP-3: metalloproteinase 3; *Ntrk2*: neurotrophin tyrosine kinase receptor 2; *Slc17a6*: vesicular glutamate transporter.

## Data Availability

The original contributions presented in this study are included in the article. Further inquiries can be directed to the corresponding authors.

## Abbreviations

The following abbreviations are used in this manuscript:

*Abcb1a*	ABC transporter subfamily B member 1a
*Abcb1b*	ABC transporter subfamily B member 1b
AGS	audiogenic seizures
ASMs	antiseizure medications
AUK	audiogenic kindling
AUK-20	20 audiogenic kindling
β-act	beta actin
BBB	blood-brain-barrier
COX-2	cyclooxygenase 2
DAB	3,3-diaminobenzidine tetrahydrochloride
Epo-R	erythropoietin receptor
ERK	extracellular signal-regulated kinase
Gabr-b1	beta 1 subunit of the GABA-A receptor
GASH/Sal	Genetic Audiogenic Seizure Hamster, Salamanca
GASH AUK	GASH/Sal subjected to AUK
GASH sAUK	GASH/Sal subjected to sustained audiogenic kindling
GASH naïve	GASH/Sal with no stimulations
GEPR	genetically epilepsy-prone rat
GTCS	generalized tonic–clonic seizures
HIF-1α	hypoxia-inducible factor 1 alpha subunit
IHC	immunohistochemistry
KM	Krushinsky-Molodkina
MDR	multidrug resistant
MMP-3	metalloproteinase 3.
NFkB	nuclear factor kappa-B transcription factor
Ntrk2	neurotrophin receptor tyrosine kinase 2
P-gp	P-glycoprotein
RE	refractory epilepsy
SD	spontaneous death
SE	status epilepticus
SS	seizure severity
SSI	seizure severity index
TLE	temporal lobe epilepsy
TrkB	tyrosine kinase receptor B
VGLUT1	vesicular glutamate transporter 1
VGLUT2	vesicular glutamate transporter 2
WAR	Wistar audiogenic rat
WB	Western blot
